# ANCA-associated vasculitis with cardiac valve vegetations in two teenage males: two case reports and a literature review

**DOI:** 10.1186/s12969-022-00750-2

**Published:** 2022-10-28

**Authors:** Alexandra Theisen, Carrie L. Phillips, Martha Rodriguez

**Affiliations:** 1 Pediatric Rheumatology, Riley Hospital for Children, Indiana University School of Medicine, 1200 West Michigan Street Suite 201, Indianapolis, Indiana 46202 USA; 2 Pediatric Rheumatology, Saint Louis University, SSM Health Cardinal Glennon Children’s Hospital, 1465 South Grand Boulevard, Glennon Hall RM 3713, Saint Louis, Missouri 63104 USA; 3 Department of Pathology, Indiana University School of Medicine, Indianapolis, Indiana 46202 USA

**Keywords:** Valvular involvement, Granulomatosis with Polyangiitis, Pediatrics, Adolescent, ANCA-associated Vasculitis

## Abstract

**Background:**

Anti-neutrophil cytoplasm antibody (ANCA)-associated vasculitis is a term used to describe systemic vasculitides that affect small and medium-sized blood vessels. Granulomatosis with Polyangiitis (GPA), a type of ANCA-associated vasculitis (AAV), is rare in children with an estimated prevalence of 3–4 per million, and even more rare is the manifestation of cardiac abnormalities secondary to ANCA-associated vasculitis in the pediatric population.

**Case presentation:**

We discuss the cases of two teenage males who presented with cardiac valvular lesions secondary to GPA in addition to sinus, pulmonary, renal, and cutaneous involvement. These findings of cardiac valvular abnormalities in GPA have rarely been described in the literature in pediatrics. Both patients were treated with rituximab, high-dose methylprednisolone, and therapeutic plasma exchange and showed improvement in their disease manifestations.

**Conclusions:**

A review of the literature revealed only five pediatric cases of ANCA-associated vasculitis with cardiac manifestations, and interestingly, three of the five had valvular involvement. Subsequent valvular involvement makes obtaining the diagnosis of ANCA-Associated Vasculitis difficult due to concern for underlying infectious endocarditis and can lead to misdiagnosis given the rarity of cardiac involvement in ANCA-associated vasculitis. Routine echocardiogram is not always completed in newly diagnosed AAV, yet cardiac involvement can lead to severe consequences as was seen with our first patient in the form of thromboembolic stroke. We discuss the importance of keeping AAV on the differential when cardiac lesions are present as well as the importance of regular cardiac screening in newly diagnosed patients with AAV, as it is a major factor of cardiac morbidity and mortality in the adult population and can contribute substantially to management decisions.

## Background

Granulomatosis with Polyangiitis (GPA), a type of anti-neutrophil cytoplasm antibody (ANCA)-associated vasculitis (AAV), is a primary systemic vasculitis involving small and medium-sized blood vessels that is characterized by inflammation within blood vessel walls, causing eventual tissue ischemia and necrosis [1, 2]. AAV is extremely rare in childhood, with GPA recognized as the most common AAV subtype present in pediatrics, followed by Microscopic Polyangiitis (MPA) and Eosinophilic GPA (EGPA) [2, 3]. Cardiac involvement, while more common in EGPA, is a rare manifestation of GPA and MPA and is estimated to affect only 5% of pediatric GPA and MPA cases [4, 5]. When cardiac involvement is present, pericarditis and/or conduction abnormalities are the typical manifestations based on adult studies, while valvular vegetations are considered rare [6, 7].

The diagnosis of GPA is based on a combination of factors, including clinical features, serological markers, and characteristic findings on biopsy. A classification criteria by the EULAR/Pediatric Rheumatology International Trials Organization (PRINTO)/Pediatric Rheumatology European Society (PReS), a pediatric-specific adaptation of American College of Rheumatology criteria, was developed using data from children; however, these criteria do not include cardiac manifestations given their rarity [8] (Table 1). Cardiac involvement is extremely rare in GPA in both adults and children. In the largest cohort of children with a diagnosis of GPA (ARChiVe Cohort, *n* = 183), only 5% of children (*n* = 10) had cardiovascular manifestations [9]. These cardiac manifestations were primarily venous thromboses; other manifestations (specifically valvular abnormalities) were not described [9]. Cardiac evaluation is not routinely completed in patients with suspected GPA or MPA who are asymptomatic from a cardiac standpoint upon presentation. When cardiac involvement is found in patients with elevated ANCA titers, the diagnosis of AAV becomes difficult as ANCA-associated infective endocarditis has been well-described in the literature and has been reported to mimic the clinical manifestations of AAV [10–17].

**Table 1:** EULAR/PRINTO/PReS Criteria for Childhood Granulomatosis with Polyangiitis

A patient is said to have GPA when three of the following six criteria are present:	
Upper Airway Involvement	Chronic purulent or bloody nasal discharge, or recurrent epistaxis/crusts/granulomata Nasal septal perforation or saddle-nose deformity Chronic or recurrent sinus inflammation
Pulmonary Involvement	Chest X-ray or CT scan showing the presence of nodules, cavities, or fixed infiltrates
Renal Involvement	Proteinuria > 0.3 g/24H, or greater than 30 umol/mg of urine albumin/creatinine ratio on a spot morning sample Hematuria or red blood cell casts: > 5 red blood cells per high-power field, or red blood cell casts in urinary sediment, or > 2+ on dipstick Necrotizing pauci-immune glomerulonephritis
Granulomatous Inflammation	Granulomatous inflammation within wall of artery or in perivascular or extravascular area of artery or arteriole
Laryngotracheobronchial stenosis	Subglottic, tracheal, or bronchial stenosis
ANCA	ANCA positivity by immunofluorescence or by ELISA (MPO/p or PR3/c ANCA)

*ANCA* Antineutrophil cytoplasmic antibody, *CT* Computed tomography, *ELISA* Enzyme-linked immunosorbent assay, *EULAR* European League Against Rheumatism, *GPA* Granulomatosis with polyangiitis, *MPO* Myeloperoxidase, *PRINTO* Pediatric Rheumatology International Trials Organization, *PR3* Proteinase 3, *PReS* Pediatric Rheumatology European Society

Here, we present two cases of pediatric GPA with significant cardiac involvement in the form of valvular vegetations, which were accompanied by pulmonary and renal injury. We review similar cases described in the literature, which have rarely included reports of cardiac valvular abnormalities in children. We emphasize the importance of an initial cardiac evaluation and regular routine cardiac monitoring in children diagnosed with AAV. We endorse the need to include AAV in the differential diagnosis in the context of cardiac abnormalities and the absence of an identifiable infection.

## Case presentations

### Case 1

**Fig. 1:**
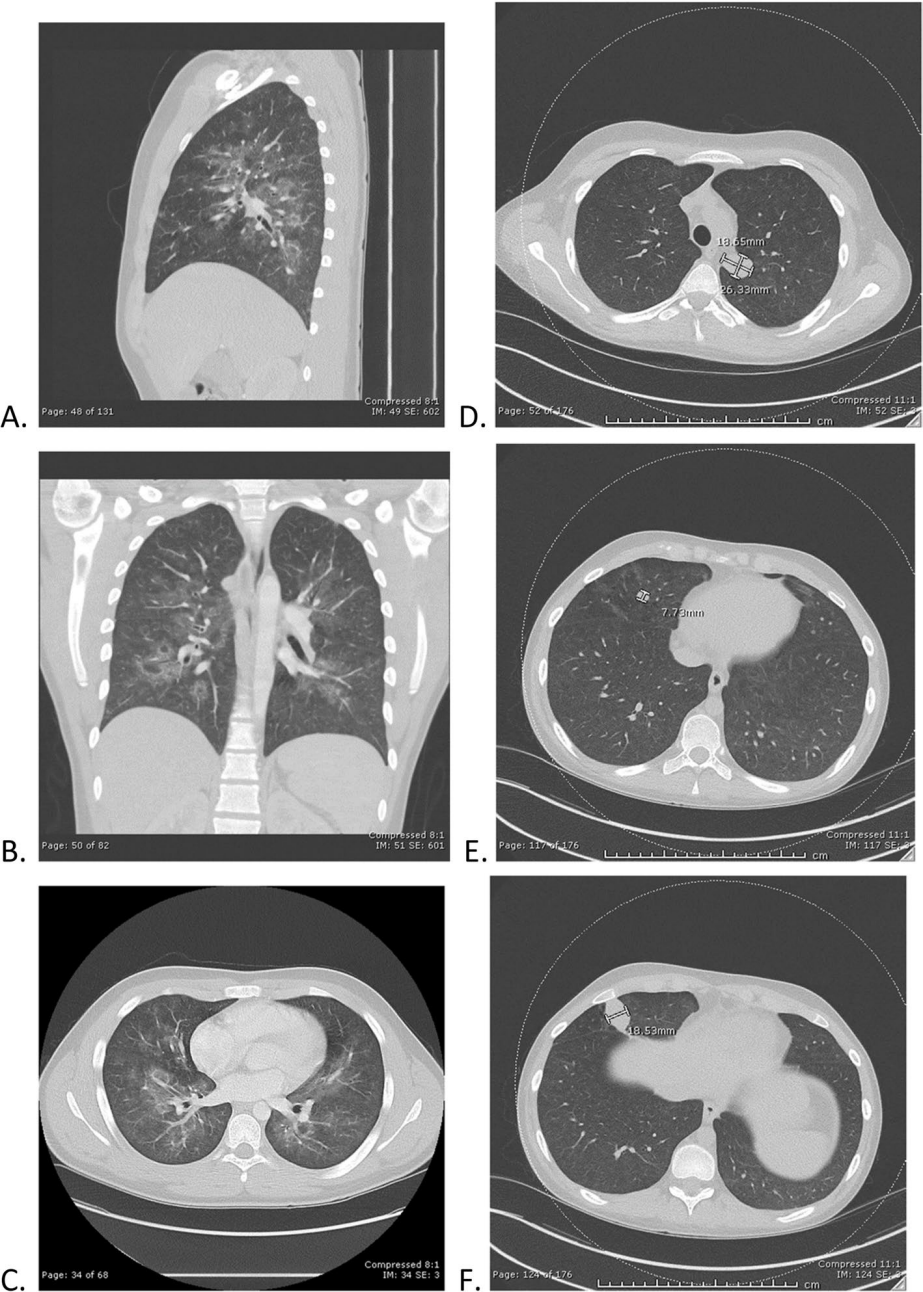
CT scan findings for patient 1 (**A-C**) and patient 2 (**D**-**F**). **A**, **B**, **C**: CT chest of Patient 1. Final read: Ill-defined, confluent groundglass attenuation is present bilaterally in the lungs, roughly following the bronchovascular bundles. No dense consolidation is seen. No pleural effusions are identified. **D**, **E**, **F**: CT Chest of Patient 2. Final read: Subtle diffuse symmetric ground glass opacities throughout both lungs. Findings may represent pulmonary hemosiderosis related to sequela of prior diffuse bilateral pulmonary hemorrhage. Two well defined solid appearing soft tissue density nodules within the right middle lobe with surrounding tiny parenchymal cysts are present. Another similar appearing well defined nodule centered in the upper aspect of the major fissure of the left lung where it contacts the mediastinum is present. These nodular opacities are without cavitation or calcification and were not clearly seen on prior portable chest radiographs. Evaluation is limited due to the lack of IV contrast and their etiology is unclear. The tiny parenchymal cysts adjacent to the nodules in the right middle lobe could represent sequela of a necrotizing lung process. These nodules could represent organized parenchymal or pleural hematomas. The appearance is not highly suggestive of infection due to lack of surrounding inflammation. Neoplasm seems unlikely given the history

**Fig. 2:**
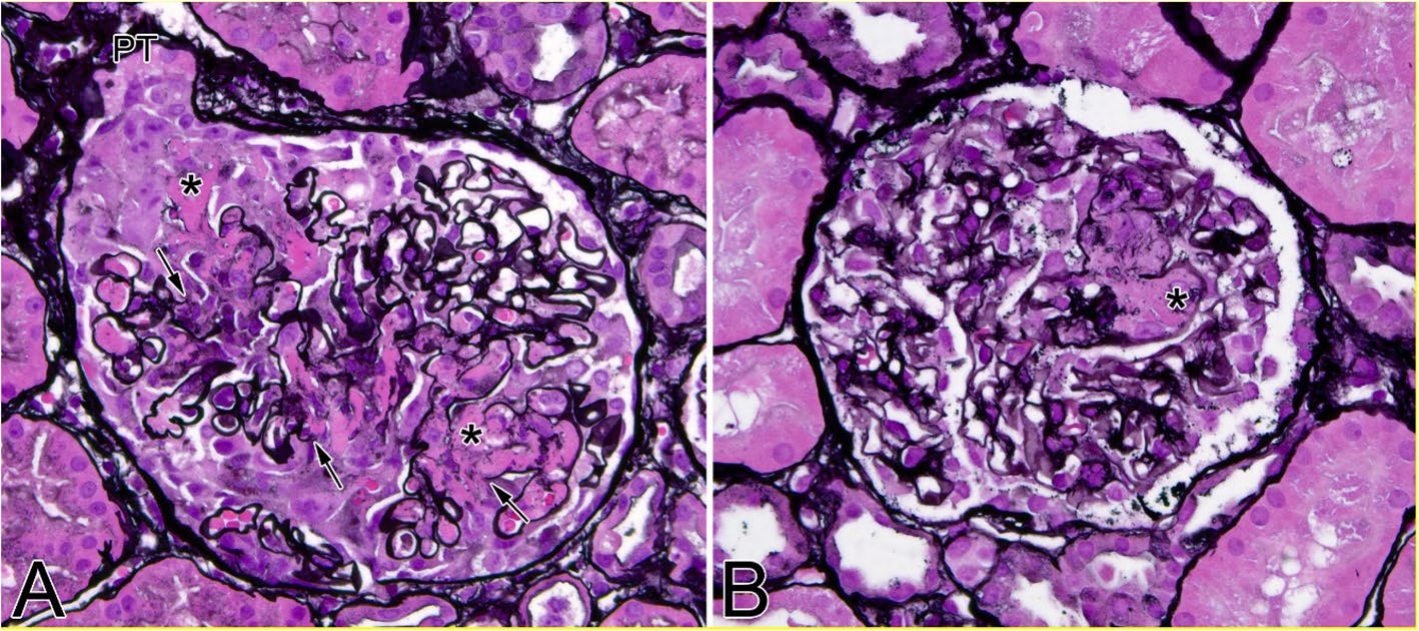
Renal histology photomicrographs for patient 1 (**A**) and patient 2 (**B**). Light microscopy: Paraffin tissue sections from formalin-fixed kidney biopsy specimens were stained with Jones’ silver. Panel A from patient 1 shows a representative glomerulus with segmental fibrinoid necrosis and early cellular crescent formation (extracapillary hypercellularity is most pronounced at urinary pole near proximal tubule/PT). Panel B from patient 2 shows segmental fibrinoid necrosis in a single glomerulus, which was not accompanied by a crescent. In both panels the arrows point to ruptured glomerular capillary loop basement membranes. Fibrin tactoids are marked with asterisks. Original magnification 40x

**Fig. 3:**
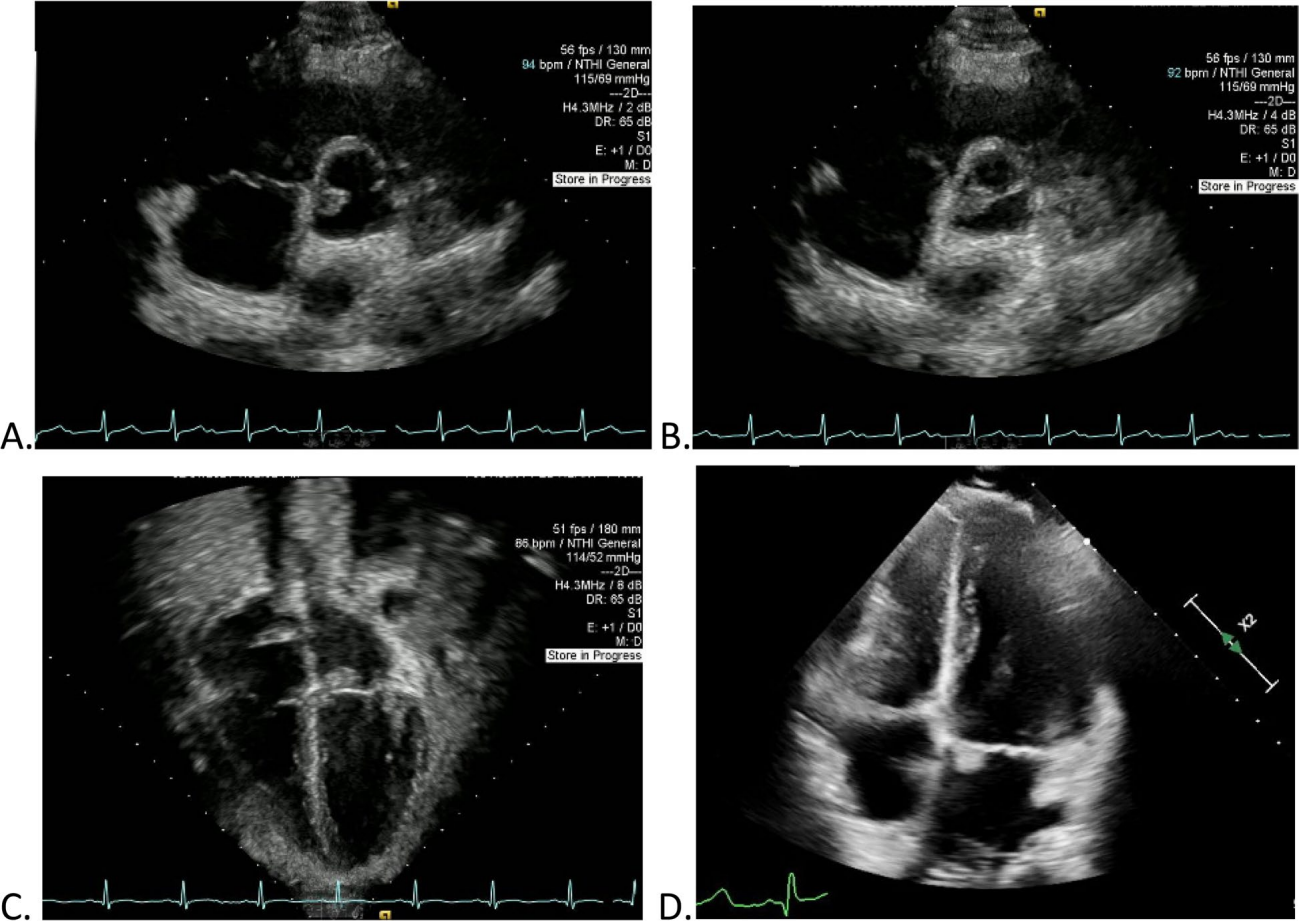
Echocardiogram abnormalities for patient 1 (**A**, **B**) and patient 2 (**C**, **D**). **A**, **B** Demonstration of Aortic Valve Vegetation for Patient 1. **C**, **D** Demonstration of Mitral Valve Vegetation for Patient 2

A 15-year-old male presented to our pulmonary service with a 6-week history of bloody noses, hemoptysis, cough, fatigue, weight loss, fevers, and dark urine. He denied cardiac symptoms and was without palpitations, chest pain, or chest tightness. He had hypertension with blood pressures in the range of 120–166 / 44–92 mmHg, hemoglobinuria (large) with red blood cells (RBC) > 100 per high-powered field (hpf), proteinuria (100 mg per deciliter (mg/dL)) with urine protein to creatinine ratio of 2.07, erythrocyte sedimentation rate (ESR) 59 mm per hour (mm/hr), C-reactive protein (CRP) 12.9 mg/dL, and hemoglobin 7.3 g per deciliter (GM/dL) (Table 2). He was intermittently febrile with a maximum body temperature of 38.8 degrees Celsius. His serum creatinine peaked at 0.98 mg/dL during his hospital stay. His chest CT showed widespread bilateral ill-defined ground-glass opacities along the bronchovascular bundles (Fig. 1). Rheumatology, Nephrology, and Infectious Disease services were consulted. Serum ANCA showed a cytoplasmic (C-ANCA) pattern with a titer of 1:640 and Proteinase-3 (PR3) value of 812 AU/mL. Additional rheumatologic laboratory tests returned normal/negative. A thorough evaluation for infection on admission was unremarkable (Table 2). On hospital day 3, he underwent renal biopsy due to persistent hypertension and elevated urine protein/creatinine ratio. The renal biopsy specimen showed focal necrotizing and crescentic glomerulonephritis (14/34 glomeruli with active lesions, see Fig. 2). Immune complex deposits were not seen in glomeruli by immunofluorescent and electron microscopy (i.e., pauci-immune), compatible with AAV. During his renal biopsy, he incidentally was found to have Mobitz II heart block on telemetry, which ultimately lead to his cardiac evaluation and cardiology consultation. Of note, he had an EKG on admission that was normal. His echocardiogram obtained on hospital day 3 showed thickened aortic valve leaflets with perforation within the right coronary leaflet, one to two small areas with vegetation on the aortic valve with mild aortic regurgitation and thickening of the anterior leaflet of the mitral valve with Ejection Fraction 60% (Fig. 3). He remained asymptomatic without palpitations, chest pain, or chest tightness. On hospital day 3, after completion of his echocardiogram, he experienced an acute ischemic event that resulted in left facial droop and left-sided hemineglect with abnormal sensations on his left side. Magnetic Resonance Angiogram (MRA) confirmed moderate-sized acute infarct involving the right insula, adjacent frontoparietal operculum and right parietal lobe with scattered foci of punctate infarcts bilaterally, suggesting thromboembolic phenomenon. Due to concern for bacterial endocarditis with newfound valvular lesions, blood cultures were drawn. Greater than 24 hours after collection, one bottle out of four grew coagulase-negative Staphylococcus, a common contaminant (Table 2). He received therapeutic plasma exchange for five days and was treated with Rituximab 1000 mg (mg) at week 0 and week 2 per ANCA-Vasculitis protocol, and methylprednisolone pulse (30 mg per kilogram (mg/kg) × 3 days, maximum 1000 mg) followed by high-dose oral glucocorticoids 2 mg/kg/day [18]. He was initiated on lisinopril 10 mg daily for hypertension, improvement in heart muscle function, and prevention of cardiac remodeling. Due to inability to exclude bacterial endocarditis based on one positive blood culture and findings on echocardiogram of valvular vegetations, he was also treated for 4 weeks with Ceftriaxone. Six months post-induction with Rituximab he was asymptomatic and displayed normalization of inflammatory markers and ANCA serology, resolution of proteinuria and hematuria, resolution of his aortic valve vegetation with stable mild-moderate aortic valve regurgitation with perforation, and no neurological or cognitive deficits.

**Table 2:** Lab Results for Case 1 and Case 2 during hospitalization(s)

**Laboratory Investigations: General**	**Laboratory Reference Ranges**	**Case 1 Laboratory Results**	Case 2 Laboratory Results
Erythrocyte Sedimentation Rate (ESR)	0–15 mm/hr	59 mm/hr at presentation; 2 mm/hr 6 months post-treatment	14 mm/hr at presentation; 2 mm/hr 6 months post-treatment
C-reactive Protein (CRP)	< 0.5 mg/dL	12.9 mg/dL at presentation; < 0.5 mg/dL 6 months post-treatment	25 mg/dL at presentation; < 0.5 mg/dL 6 months post-treatment
Hemoglobin	13.0–16.0 g/dL	7.3 GM/dL	8.3 GM/dL
Creatinine	0.71–1.16 g/dL	0.98 at presentation	1.21 at presentation
Urinalysis	RBC: 0–2/hpf Protein: Negative	RBC > 100/hpf Protein 100 mg/dL	RBC 6–10/hpf at admission; > 100/hpf day 9 hosopitalization
Protein/Creatinine ratio	0- < 0.2	2.07	3.72
Laboratory Investigations: Rheumatologic			
Anti-Nuclear Antibody (ANA)	< 1:80	1:80	< 1:80
Double-Stranded DNA	0–9 IU/mL	0 IU/mL	0 IU/mL
Complements (C3, C4)	C3: 82–193 mg/dL C4: 15–57 mg/dL	C3: 154 mg/dL C4: 32 mg/dL	C3: 169 mg/dL C4:26 mg/dL
Anti-Smith	0.0–0.9	< 0.2	< 0.2
anti-RNP	0.0–0.9	< 0.2	< 0.2
SSA	0.0–0.9	< 0.2	< 0.2
SSB	0.0–0.9	< 0.2	< 0.2
Cardiolipin IgG	< 20 GPL U/mL	0 GPL U/mL	0 GPL U/mL
Cardiolipin IgM	< 20 MPL U/mL	0 MPL U/mL	0 MPL U/mL
Beta-2-Glycoprotein IgG	< 20 SGU	Not detected	Not detected
Beta-2-Glycoprotein IgM	< 20 SMU	Not detected	Not detected
DRVVT	33–43 seconds	35 seconds	38 seconds
STACLOT	No lupus anti-coagulant detected	No lupus anti-coagulant detected	No lupus anti-coagulant detected
Anti-glomerular basement membrane antibody IgG (EU)	0–19 AU/mL	0 AU/mL	0 AU/mL
ANCA profile (during hospitalization)	< 1:20 AU/mL, PR3 0	C-ANCA pattern 1:640, PR3 812 AU/mL	c-ANCA pattern 1:5120, PR3 1242 AU/mL
ANCA profile (6 months post-treatment)	< 1:20 AU/mL, PR3 0	< 1:20 AU/mL, PR3 0	< 1:20 AU/mL, PR3 0
Laboratory Investigations: Infectious			
Hepatitis A Antibodies, total Hepatitis A Antibody, IgM	Negative	Negative	Negative
Hepatitis B Virus Core Antibodies (total) Hepatitis B Virus Core Antibody, IgM Hepatitis B Virus Surface Antibody Hepatitis B Virus Surface Antigen	Negative	Negative	Negative
Hepatitis C Virus Genotype Hepatitis C Virus Antibody Hepatitis C Virus by Quantitative NAAT	Negative	Negative	Negative
*Bartonella henselae* Antibody, IgG and IgM	Negative	Negative	Negative
*Bartonella quintana* Antibody, IgG and IgM	Negative	Negative	Negative
Herpes Simplex Virus (HSV) DFA with Reflex to HSV Culture	Negative	Negative	Negative
Blood cultures	Negative	1 of 4 bottles positive for Coagulase-Negative Staphylococcus > 24 hours after collection	Negative
PCR Respiratory Viral Panel^a^	Negative	Negative	Negative
AFB Stain and culture: Bronchoalveolar lavage (BAL)	No organisms on gram stain; negative cultures	Negative	Negative
Fungal stain and culture: BAL	No organisms on gram stain; negative cultures	Negative	Negative
Bacterial stain and culture: BAL	No organisms on gram stain; negative cultures	Negative	Negative
Universal PCR: BAL	Negative	Negative	Negative
Aspergillus antigen Index: BAL	< 0.5	< 0.5	0.55
Anti-Streptolysin O	0–240 IU/mL	125 IU/mL	200 IU/mL
DNAse B	0–250 U/mL	< 250 U/mL	< 250 U/mL
Tuberculosis	Negative	Negative	Negative
Histoplasmosis antigen (urine)	< 0.5 ng/mL	< 0.5 ng/mL	< 0.5 ng/mL
Histoplasmosis antigen (serum)	0.19–60.0 ng/mL	Not detected	Not detected
Human Immunodeficiency Virus (HIV)	Negative	Negative	Negative

^a^Respiratory Viral Panel: PCR swab for Parainfluenza, Respiratory Syncytial virus (RSV), *Bordetella parapertussis*, *Bordetella pertussis*, *Chlamydia pneumoniae*, *Mycoplasma pneumoniae*, COVID-19, Human Metapneumovirus, Human Rhinovirus/Enterovirus, Adenovirus, Coronavirus 229E, Coronavirus HKU1, Coronavirus NL63, Coronavirus OC43, Influenza A and Influenza B

### Case 2

A 16-year-old male was admitted to the Intensive Care Unit (ICU) due to acute hypoxic respiratory failure. He had a history of stiff, painful knees and elbows 3 weeks prior that had resolved with non-steroidal anti-inflammatory (NSAID) use. One week prior, he presented to an outside hospital due to cough, congestion, fevers, and myalgias. At this visit, his chest radiograph showed findings suspicious for pneumonia; however, his symptoms did not improve despite Azithromycin therapy. He was evaluated by his primary care physician who tested him for COVID-19 (negative) and Influenza A (positive). He was given Amoxicillin and Doxycycline due to concern for overlying bacterial pneumonia, however he then developed weakness and severe shortness of breath. He presented to outside emergency department where he was found to be febrile to 38.0 degrees Celsius and tachycardic, with blood oxygen saturations of 65% while on room air. He had elevated white blood cell count to 18.5 k/cumm, hemoglobin 8.3 mg/dL, serum creatinine 1.21 mg/dL, blood urea nitrogen (BUN) 31 mg/dL, mild coagulopathy, CRP 25 mg/dL, ESR 14 mm/hr. and urinalysis significant for hemoglobinuria with 6–10 RBC/hpf (Table 2). Chest radiograph showed the development of large bilateral pneumothoraces with pneumomediastinum and dense opacities in both lungs. He required high-flow nasal cannula but soon transitioned to Bilevel positive airway pressure (BiPAP) and ultimately intubation. On day 2 he was transferred to our ICU and placed on Venovenous (VV) extracorporeal membrane oxygenation (ECMO) due to severe respiratory failure secondary to pulmonary hemorrhage confirmed by bronchioalveolar lavage (BAL). An echocardiogram completed on admission showed a probable thrombus/vegetation on the mitral anterior leaflet near the septal attachment, trivial mitral regurgitation with normal tricuspid valve, and ejection fraction of 67%. Cardiology was immediately consulted. Due to clinical instability, a transesophageal echocardiogram was contraindicated. Repeat echocardiogram on day 8 of hospitalization confirmed the presence of the vegetation in addition to perforation of one of the anterior leaflets of the mitral valve. He was initiated on broad spectrum antibiotics (Cefepime, Gentamicin, and Vancomycin) upon admission due to concern for overlying bacterial pneumonia and infectious endocarditis. The Pediatric ICU team began methylprednisolone 2 mg/kg/day from day 2 through day 4 of hospitalization due to diffuse pulmonary hemorrhage, significant acute respiratory distress syndrome, and requirement for VV ECMO. While on IV methylprednisolone, his serum creatinine improved and his VV ECMO settings were decreased, though both renal and lung function slowly worsened after discontinuation of steroids. Infectious Disease, Rheumatology, and Nephrology services were consulted on days 6, 9, and 12 of hospitalization, respectively.

A thorough evaluation for infection ensued on day 6 of hospitalization, which was unremarkable aside from slightly positive Aspergillus antigen in his BAL (Table 2), though our Infectious Disease colleagues doubted this was a true-positive. On hospital day 6, a chest CT-Angiogram was completed to evaluate the extent of thromboembolic phenomenon given cardiac vegetations, which showed diffuse consolidation of both lungs without evidence of pulmonary arterial embolic disease. On hospital day 6, he developed splinter hemorrhages, followed by scattered petechiae on hospital day 7 consistent with leukocytoclastic vasculitis that was confirmed by skin biopsy. Repeat echocardiogram on hospital day 8 re-demonstrated small mitral valve vegetation and likely perforation of the anterior leaflet near the medial annulus (Fig. 3)**.** Rheumatology was consulted on day 9 of hospitalization due to worsening hematuria (RBC on admission 6–10/hpf, hospital day 9 with RBC now > 100/hpf) with large hemoglobinuria and worsening urine protein/creatinine ratio (now 3.72), pulmonary hemorrhage, leukocytoclastic vasculitis, absence of a detectable source of infection (Table 2), and vegetations on echocardiogram with concern for an underlying rheumatologic disorder.

Rheumatologic evaluation was unremarkable aside from c-ANCA pattern with titer of 1:5120 and PR3 1242 AU/mL (Table 2). Results of his ANCA panel drawn on hospital day 9 were received on hospital day 14, by which time his renal function had deteriorated. He required continuous venovenous hemofiltration (CVVH) initiated on hospital day 11. He remained on VV ECMO, which precluded a percutaneous needle biopsy of the kidney due to risk of hemorrhage. Pulse methylprednisolone (500 mg q12 for a total of 3 g) and a five-day course of therapeutic plasma exchange were initiated on hospital day 11 due to rapidly progressing rash, worsening renal function and hyperkalemia requiring CVVH, negative testing for infection, pulmonary hemorrhage, and risk of renal biopsy. Upon initiation of pulse methylprednisolone and therapeutic plasma exchange, his lung function and kidney function improved. He was decannulated from VV ECMO on hospital day 20 and discontinued CVVH on hospital day 19. A renal biopsy specimen obtained on hospital day 22 showed focal necrotizing glomerulonephritis (1/23 glomeruli with activity, see Fig. 2) in the absence of detectable crescents. Immune complex deposits were not seen in glomeruli by immunofluorescent and electron microscopy (i.e., pauci-immune), compatible with AAV. When stabilized, he underwent a CT scan of his chest which showed diffuse symmetric ground glass opacities bilaterally and two well-defined solid appearing soft tissue density nodules in the right middle lobe and another nodule in the upper aspect of the major fissure of the left lung (Fig. 1). Collectively, these findings are consistent with GPA.

For GPA, he received high-dose pulse glucocorticoid for 3 days (hospital day 11–13) followed by high-dose oral glucocorticoids (prednisone 30 mg twice daily), therapeutic plasma exchange for 5 consecutive days (hospital day 11–15), and ultimately Rituximab 375 mg per meter-squared (mg/m2) every week (first dose on hospital day 30) for a total of four doses per protocol followed by 1000 mg every 6 months [18]. Due to our inability to exclude infectious endocarditis despite negative blood cultures, he completed 4 weeks of Ceftriaxone. Infectious disease team initiated treatment for Aspergillus due to detection of Aspergillus antigen in BAL and known need for immunosuppression with B-cell depleting therapy. Due to persistent hypertension, proteinuria, and risk of cardiac remodeling, we initiated lisinopril 10 mg daily per recommendations of Cardiology and Nephrology consultants. He clinically improved. At 6 months post-induction therapy he received maintenance therapy with Rituximab 1000 mg every 6 months, with baseline serum creatinine of 1.5 mg/dL, resolution of hematuria and proteinuria, improved lung function, and resolution of mitral valve vegetation with stable mild mitral regurgitation and persistent perforation within the anterior mitral valve leaflet.

## Discussion

These two cases highlight significant valvular involvement in GPA in two teenage male patients, an extremely rare finding in the pediatric literature. Based on our literature search, only five other patients within the pediatric age realm have been described as having cardiac involvement **(**Table 3). Interestingly, three of the five patients had cardiac valvular abnormalities, which suggests that valvular abnormalities, while rare, require consideration in the pediatric age group. In adults, the most prominent valve abnormalities reported in one study of patients with EGPA and GPA was the aortic valve in the form of regurgitation [24]. Our first patient did have aortic valve vegetations, and aortic valve involvement has been reported in one case of AAV in children [22] (Table 3). Our second patient had mitral valve involvement with GPA, which has not previously been reported in children. The other pediatric reports describe two cases of tricuspid valve involvement, one case of ventricular involvement, and one case of aneurysm formation [19–21, 23] (Table 3). Our two patients, plus the five published cases, had other systemic features suggestive of AAV, for a total of seven patients. Five of these seven patients had evidence of c-ANCA pattern with PR3 antigen positivity. The outcomes of patients with cardiac involvement in AAV is unclear, though out of the seven cases, four showed improvement upon implementation of appropriate immunosuppressive therapy (Table 3). Interestingly, the ARCHiVe cohort, the largest cohort of pediatric patients diagnosed with GPA described in the literature, found no cardiovascular manifestations at presentation in 65 patients [4]. A follow-up study demonstrated only 10 patients out of 183 with cardiovascular manifestations [9], while a separate cohort described zero patients out of 38 [5]. Our results, combined with these published studies, highlight the rarity of cardiac manifestations in GPA in children.

**Table 3:** Pediatric Cases of ANCA-Vasculitis Associated with Cardiac Involvement

Author	Patient Age, Sex	Onset of Cardiac Involvement	Description of Cardiac Involvement	Other organ involvement	ANCA-titer at time cardiac involvement found (pattern and antigen)	Treatment	Outcome
N/A	15yo, M	Diagnosis	thickened aortic valve leaflets with perforation within the right coronary leaflet as well as 1–2 small areas with vegetation on the aortic valve with mild aortic regurgitation, as well as thickening of the anterior leaflet of the mitral valve	Upper and lower respiratory tract, renal, cutaneous, CNS (thromboembolic stroke)	C-ANCA pattern 1:640, PR3 812 AU/mL	Pulse glucocorticoids (1GMx3 days), 5 days TPE, followed by methylprednisolone 2 mg/kg/day, Rituximab 1000 mg week 0 and week 2, followed by 1000 mg Rituximab every 6 months	Normalization of renal function, resolution of inflammatory markers and ANCA titers, recovery of symptoms; ultimately remission on treatment
N/A	16yo, M	Diagnosis	small mitral valve vegetation, likely perforation of the anterior leaflet near the medial annulus	Lower respiratory tract, cutaneous, renal	c-ANCA pattern 1:5120, PR3 1242 AU/mL	Pulse glucocorticoids (1GMx3 days), 5 days TPE, followed by methylprednisolone 2 mg/kg/day, Rituximab 375 mg/m2 once weekly for 4 doses, followed by 1000 mg Rituximab every 6 months	Improvement in renal function, resolution of inflammatory markers and ANCA titers, recovery of symptoms; ultimately remission on treatment
Harris et al. [19]	14yo, F	Diagnosis	Ovoid, homogenous pedunculated mass in apex of left ventricle with EF 40%	Upper and lower respiratory tract, cutaneous, arthritis, renal	C-ANCA (titer not available); PR3 148 EU/mL	Pulse glucocorticoids (30 mg/kg) followed by methylprednisolone 2 mg/kg/day, cyclophosphamide 1 mg/kg/day, 7 days TPE; mass resection	Unknown
Kosovsky et al. [20]	16yo, M	Diagnosis	Ventricular tachycardia, mass involving full thickness of right anterior ventricular wall extending into base of papillary muscles of the anterior leaflet of the tricuspid valve; biopsy revealed acute and organizing granulomatous vasculitis	Upper and Lower respiratory tract	ANCA studies negative	Cyclophosphamide (exact dosing not provided), Prednisone (exact dosing not provided)	Unknown
Varnier et al. [21]	16yo, M	Diagnosis	Vegetation adjacent to tricuspid valve	Cutaneous, upper and lower respiratory tract, renal, genital	C-ANCA (titer not available); PR3 194 EU/mL	Methylprednisolone pulse 1 g daily for 3 consecutive days followed by high dose oral prednisolone; 10 days TPE; Rituximab 1 g at week 0 and 2; Cyclophosphamide 500 mg/m2 every 3 weeks for 4 doses	Normalization of renal function, resolution of inflammatory markers, recovery of symptoms; ultimately remission on treatment with prednisolone 3 mg daily, Azathioprine 125 mg daily, Amlodipine 5 mg daily
Leff et al. [22]	17yo, M	Diagnosed 1 year after treatment	Mild LV enlargement, AI, AV perforation	Cutaneous, upper and lower respiratory tract, arthritis	C-ANCA 1:512; PR3 (exact unavailable)	Oral cyclophosphamide 150 mg/day, prednisone 40 mg/day	AV replacement due to progressive insufficiency; flare -- > re-initiation of cyclophosphamide, oral prednisone, Bactrim
Moghadam et al. [23]	10yo, M	Diagnosis	Diffuse ectasia and dilation with large aneurysm in the left anterior descending artery (14 mm)	Perforated otitis media with effusion, saddle nose deformity, mastoiditis, sinusitis, deep vein thrombosis	P-ANCA (titer not available); antigen not provided	Methylprednisolone pulse (30 mg/kg/day for 3 days/monthly), cyclophosphamide 750 mg/m2/monthly for 6 months. Oral prednisolone 1 mg/kg/day, mycophenolate mofetil 1200 mg/m2/day. Aspirin 5 mg/kg, warfarin 0.1 mg/kg.	Maintenance therapy with low-dose prednisolone, mycophenolate mofetil, antithrombotic therapy. No flare 1.5 years after diagnosis.

*Abbreviations*: *EF* Ejection Fraction, *LV* Left Ventricular, *AI* Aortic Insufficiency, *AV* Aortic Valve, *TPE* Therapeutic Plasma Exchange

For our first case, cardiac involvement was incidentally found due to the presentation of Mobitz type II heart block while undergoing renal biopsy and subsequent echocardiogram that was obtained due to this conduction abnormality. This patient was asymptomatic from a cardiac standpoint, yet ultimately developed thromboembolic stroke due to his cardiac involvement. To our knowledge, this is the first report of pediatric cardiac valvular involvement leading to thromboembolic stroke in GPA. Patients with GPA can be asymptomatic yet still have signs of cardiac involvement as evidenced by electrocardiogram and echocardiogram. Cardiac involvement has been described as a strong predictor of cardiac mortality in adult studies [24]. Given the high mortality and possible consequences (i.e., thromboembolic stroke) of cardiac involvement in patients with GPA, screening echocardiograms should be performed, as it may prove beneficial to gauge disease severity and guide therapy and management decisions to prevent complications of this rare form of vasculitis.

Our second case demonstrated cardiac involvement that was initially attributed to infectious endocarditis; however, upon further evaluation we diagnosed GPA. This case was extremely challenging given the severity of the patient’s presentation and limitations to obtain tissue specimens to aid with diagnostic evaluation while on VV ECMO. Elevated ANCA titers, while strongly associated with systemic vasculitides, have also been described in other settings including drug-induced AAV and infections including bacterial endocarditis. Patients with infective endocarditis have been described as having more severe renal manifestations [10–12]. Our second patient had persistently negative blood cultures throughout his hospital stay. In patients with ANCA-associated infective endocarditis, positive blood cultures are typically more prevalent than in ANCA-negative infective endocarditis [13, 17]. B-cell depleting therapy was not initiated in our patient until infection was excluded and a percutaneous renal biopsy had been safely performed. His renal biopsy specimen was consistent with AAV based on the findings of focal necrotizing glomerulonephritis (i.e., isolated fibrinoid necrosis) in the absence of immune complex deposits in glomeruli (i.e., pauci-immune). Pauci-immune fluorescence pattern has been described in patients with ANCA-associated infective endocarditis [14, 15]. However, patients with ANCA-positive infective endocarditis typically have organ involvement limited to skin and kidneys, positive blood cultures, abnormal levels of serum complement, immune complexes deposited in tissues, and detection of other autoantibodies (i.e., rheumatoid factor, antinuclear antibodies, anticardiolipin antibodies) [13, 15, 16, 18]. Our patient did not have any of these findings and had additional lung involvement. This case highlights the diagnostic challenge in a patient with cardiac valvular vegetations, rash, fevers, pulmonary and renal involvement, and elevated ANCA titers. In such cases, it is imperative to exclude infectious etiologies and, if possible, confirm the diagnosis with biopsy prior to initiation of immunosuppression. However, this may not be feasible due to severity of clinical presentation and the need for empiric treatment with antibiotics and immunosuppression.

## Conclusions

These two cases highlight cardiac involvement in GPA in two teenage males and the significant consequences that can occur from such involvement and related diagnostic dilemmas. Cardiac valvular involvement is a rare manifestation of GPA in pediatrics, having only been previously reported in five cases, with three of the five manifesting as valvular vegetations or abnormalities. While cardiac involvement in adults has been associated with increased all-cause cardiac mortality, prognosis in children is unknown. Cardiac evaluation with echocardiogram is essential in pediatric cases of vasculitis to fully evaluate the extent of disease and to guide treatment and management decisions. When cardiac involvement is found in cases of ANCA-positivity with multi-organ involvement, a thorough evaluation for infection should be completed. Tissue biopsy should ideally be performed to both exclude an infectious process and render a diagnosis of AAV prior to initiation of immunosuppressive therapy.

## Data Availability

Not applicable.

## Abbreviations

ANCA: Anti-neutrophil cytoplasmic antibody
AAV: ANCA-Associated Vasculitis
GPA: Granulomatosis with Polyangiitis
MPA: Microscopic Polyangiitis
EGPA: Eosinophilic Granulomatosis with Polyangiitis
CVVH: Continuous Veno-Venous Hemofiltration
PR3: Proteinase 3
VV ECMO: Venovenous Extracorporeal Membrane Oxygenation
MPO: Myeloperoxidase
IV: Intravenous
IE: Infective Endocarditis
BAL: Bronchoalveolar lavage
MRA: Magnetic Resonance Angiogram
CT: Computerized tomography
